# Complex patterns shape immune genes diversity during invasion of common raccoon in Europe – Selection in action despite genetic drift

**DOI:** 10.1111/eva.13517

**Published:** 2022-12-11

**Authors:** Maciej K. Konopiński, Anna M. Fijarczyk, Aleksandra Biedrzycka

**Affiliations:** ^1^ Institute of Nature Conservation Polish Academy of Sciences Kraków Poland; ^2^ Laval University Département de Biologie Université Laval Québec Québec Canada

**Keywords:** balancing selection, common raccoon, directional selection, diversity loss, expansion, immune genes, invasion

## Abstract

Rapid adaptation is common in invasive populations and is crucial to their long‐term success. The primary target of selection in the invasive species' new range is standing genetic variation. Therefore, genetic drift and natural selection acting on existing variation are key evolutionary processes through which invaders will evolve over a short timescale. In this study, we used the case of the raccoon *Procyon lotor* invasion in Europe to identify the forces shaping the diversity of immune genes during invasion. The genes involved in the defence against infection should be under intense selection pressure in the invasive range where novel pathogens are expected to occur. To disentangle the selective and demographic processes shaping the adaptive immune diversity of its invasive and expanding populations, we have developed species‐specific single‐nucleotide polymorphism markers located in the coding regions of targeted immune‐related genes. We characterised the genetic diversity of 110 functionally important immune genes in two invasive and one native raccoon genetic clusters, each presenting a different demographic history. Despite the strong effect of demographic processes in the invasive clusters, we detected a subset of genes exhibiting the diversity pattern suggestive of selection. The most likely process shaping the variation in those genes was balancing selection. The selected genes belong to toll‐like receptors and cytokine‐related genes. Our results suggest that the prevalence of selection depends on the level of diversity, that is – less genetically diverse invasive population from the Czech Republic displayed fewer signs of selection. Our results highlight the role of standing genetic variation in adapting to new environment. Understanding the evolutionary mechanisms behind invasion success would enable predicting how populations may respond to environmental change.

## 1 INTRODUCTION

A major challenge of evolutionary ecology is to understand and predict the consequences of the current rapid environmental changes for wild populations. Species with a low adaptive potential, that is, a poor ability to respond to selection through molecular or phenotypic changes, have lower chances of persistence in a changing environment (Lande & Shannon, 1996; Merilä & Hendry, 2014). On the other hand, invasive species, which are often subjected to genetic bottleneck when a new population is established, show a high evolutionary potential that facilitates their adaptation to novel conditions and sometimes allows them to outperform native species (Dlugosch et al., 2015). Rapid adaptation is common in invasive populations (Lee, 2002; Rollins et al., 2013) and is proposed to be crucial to their long‐term success (Dlugosch & Parker, 2008). Understanding the evolutionary mechanisms behind invasion success enable to predict how populations (both native and invasive) may respond to environmental change (Barrett, 2015; Bay et al., 2018).

A range of demographic scenarios may occur during invasion, leaving different footprints in the invasive populations' genomic diversity. Introduced populations are established with variable numbers of founding individuals from potentially different source populations (Dlugosch & Parker, 2008) and many of them are initially geographically isolated. Moreover, demographic bottlenecks often accompanying the release of the species to a new environment cause stochastic changes in genetic diversity. As a result, large differences in allele frequencies or fixation of different alleles may occur across the invasive range (Dlugosch et al., 2015; Dlugosch & Parker, 2008). Independent demographic processes in the populations introduced in geographically separated locations promote further changes in allele frequencies. However, many invasive species do not exhibit a genetic bottleneck but instead maintain high levels of genetic variation in the introduced range (Dlugosch & Parker, 2008; McGoey & Stinchcombe, 2021; Uller & Leimu, 2011). This phenomenon is often explained by the mixing of individuals originating from different populations within the native range. This can increase their adaptive potential by increasing genetic variance within populations (Bock et al., 2015; Prentis et al., 2008; Rius & Darling, 2014; Roman & Darling, 2007; Verhoeven et al., 2011). Immune‐related genes involved in the defence against infection are great candidates for studying the genomic basis of evolutionary processes in invasive species (Obbard et al., 2009; Sironi et al., 2015). Since pathogens exert a strong selection pressure on their hosts, immunity‐related genes are presumed to be under selection due to host–pathogen coevolution and evolve rapidly (McTaggart et al., 2012; Schlenke & Begun, 2003). The primary target of selection in the new range is the standing genetic variation. Therefore, genetic drift and natural selection acting on existing variation are key evolutionary processes through which invaders will evolve over a short timescale (reviewed by Ardia et al., 2011). Both the genetic drift and directional selection, which favour specific variants or purifying selection, which eliminates maladaptive variants, are thought to reduce genetic variability resulting in high frequencies of resistance alleles within a population. The coevolutionary arms race between pathogens and their hosts may lead to the fixation of beneficial alleles via positive selection (Kosiol et al., 2008; Nielsen et al., 2005). In contrast, balancing selection actively maintains multiple alleles in the population and promotes increased genetic diversity. Balancing selection maintains the genetic polymorphism over extended time scales by heterozygote advantage or negative frequency‐dependent selection, but also due to different selection pressures in a spatial or temporal context, the presence or absence of infection, or by acting differently on the individuals of different sexes. Additionally, pre‐existing genetic diversity maintained by balancing selection in the species native range should also increase adaptive potential in the invasive range (Biedrzycka et al., 2019; Lee & Gelembiuk, 2008). Diverse genetic variants transferred into a new range are putative targets of positive selection, thus increasing the probability of a successful spread.

The analyses of candidate genes, mainly in human populations (Andrés et al., 2009; Quintana‐Murci & Clark, 2013) but also in other organisms such as newts (Fijarczyk et al., 2016), rodents (Lundberg et al., 2020), snails (Tennessen et al., 2015) or monkeys (Luo et al., 2012), have identified numerous immune defence genes with signatures of balancing selection. Directional selection was also proved to shape genetic diversity of candidate immune genes in multiple species, for example, *Drosophila* (Hill et al., 2019), roe deer (Quéméré et al., 2015) or mallard ducks (Chapman et al., 2016). The analysis of the genome‐wide variation of invasive populations also identified numerous loci evolving under positive selection (Bernardi et al., 2016; Hodgins et al., 2013; Vera et al., 2016). Undoubtedly, the best‐documented example for balancing selection is the major histocompatibility complex in vertebrates (Bernatchez & Landry, 2003; Radwan et al., 2020; Spurgin & Richardson, 2010). The second group of genes that shows high levels of balanced polymorphism in natural populations are toll‐like receptors (Kloch et al., 2018; Kloch & Biedrzycka, 2020; Quéméré et al., 2015) and cytokine genes (Downing et al., 2010; Turner et al., 2012). The footprint of balancing selection has also been detected in viral resistance genes (Ferguson et al., 2008; Fish & Boissinot, 2015; Newman et al., 2006) and antimicrobial peptides (Chapman et al., 2016; Hellgren & Sheldon, 2011; Tennessen & Blouin, 2008).

In this study, we used the case of the raccoon's, *Procyon lotor*, invasion in Europe to characterise the diversity of immune genes and identify the forces shaping this diversity during the invasion. The raccoon is a medium‐sized carnivore whose native distribution in North America extends from southern Canada to Panama (Zevelhof, 2018). Its first successful introduction in Europe occurred in Germany in the 1930s with a limited number of individuals (Jernelöv, 2017). Genetic analyses of a large set of microsatellite loci and mitochondrial DNA based on a whole‐country sampling suggest that the current German population of raccoon was established by at least four small‐scale, initial independent introduction events (Fischer et al., 2017). It has been estimated that 1,000,000 raccoons are currently living in Germany, and the range of the species in Europe has extended to the west, east and south of the invasion core (Lutz, 1984; Salgado, 2018). The analysis of major histocompatibility class II gene diversity (MHC‐DRB locus), which is one of the key genes in adaptive immune response, revealed that in the invasive European population, despite the loss of many MHC alleles, functional MHC supertypes are preserved and the mean individual allele divergence remains high (Biedrzycka et al., 2019). This suggests that selection maintains divergent allele combinations despite drift causing allelic loss. The associations between specific MHC alleles and parasite infection suggest that intestinal parasites exert a selective pressure on an invasive raccoons and promote local adaptation over time (Biedrzycka, Popiołek, & Zalewski, 2020). In the invasive raccoon range, two different genetic clusters (German/Polish and Czech) have been detected based on microsatellite (Biedrzycka et al., 2014) and MHC analysis (Biedrzycka et al., 2019). The first cluster consists of a group of subpopulations stemming from the initial introductions of raccoons in Germany that have been expanding in the past few decades reaching Poland. The second cluster consists of the population from the Czech Republic, putatively established in the early 2000s by individuals that escaped from captivity. This population is isolated from other raccoon populations, has not expanded (Henryk Okarma, personal communication) and exhibits lower genetic diversity at microsatellite and MHC loci (Biedrzycka et al., 2014, 2019). Different MHC–parasite associations emerged in each cluster, altering the populations' infection status. This finding underlines the role of standing genetic variation in shaping host–parasite relationships and provides empirical support for the idea that functional genetic variation may be, at least partly, responsible for differences in the success of invasive populations (Biedrzycka et al., 2019). The third cluster included in our analysis comprises raccoons inhabiting the native population of Florida. This population also lacks signs of expansion (Troyer et al., 2014) and possesses higher levels of genetic diversity than the invasive populations (Biedrzycka et al., 2019). Although the Florida population has not been identified as a source population of raccoons introduced in Europe, it is a demographically stable population, and thus can be used as an example of a population from within the original range of the species.

To disentangle the selective and demographic processes shaping adaptive immune diversity within the three genetic clusters, we have developed species‐specific protein‐coding single‐nucleotide polymorphism (SNP) markers located in targeted immune‐related genes. We analysed the levels of genetic diversity at 110 functionally important immune genes. We aimed to: (i) compare the levels of genetic variation in clusters that experienced different demographic scenarios and (ii) search for signatures of selection in the specific genes in these clusters using tests relying on different principles. We hypothesise that: (i) the diversity of immune genes will be lower in the invasive populations than in the native Florida (FL) population, and in populations with lower level of standing genetic variation, fewer signs of selection will be visible; (ii) there will be a difference in the levels of diversity between the Czech (CZ) genetic cluster and the German/Polish (CE) genetic cluster reflecting their different demographic history and (iii) the footprint of selection should be visible despite the demographic process acting on invasive clusters since selection affects only a handful of genes.

## 2 METHODS

### 2.1 Study area and sampling methods

We collected DNA samples from the native population from Florida, USA (FL), and the two regions where raccoons were introduced in Europe: from Germany/Poland (CE, *n* = 30) and from the Czech Republic (CZ, *n* = 30). Raccoons from the native range (FL, *n* = 27) were sampled from six different localities throughout Florida. Although the samples from the native population were more scattered than those from the European populations, considering the high density of the Florida raccoon population and no substantial environmental differences, we assumed population homogeneity and similar selective pressures acting on this genetic cluster. These samples were obtained from local pest control programmes (Trujillo & Hoffman, 2017). The samples were collected between 2012 and 2017. We do not consider FL population as the source of European populations, since the origin of the latter has not yet been confirmed. We are aware that using a single native population does not allow us to draw general conclusions regarding immune genes diversity within the native range.

**FIGURE 1:**
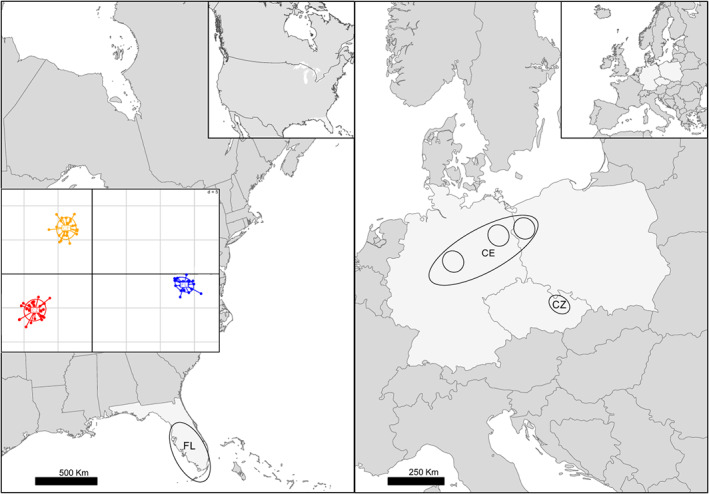
Geographical locations of native (left panel) and invasive (right panel) raccoon genetic clusters and sampling sites. Circles represent sampling locations of invasive populations and ellipses show the population grouping detected by DAPC analysis. The scatter plot presents the results of discriminant analysis of principal components (DAPC) for the genetic structure of raccoon individuals based on 427 SNPs located in 158 immune genes (inset); grid size for both x and y equals *d* = 5

The samples from the invasive clusters were muscle tissues obtained from hunters culling raccoons as part of invasive species game management activities. The geographical locations of the genetic clusters and the locations of sampling sites as well as the outcome of the genetic structure analysis are presented in Figure 1. All tissue samples were stored at −20°C prior to DNA extraction. Additionally, the spleen and liver samples from four Polish raccoons were obtained immediately after the animals were killed. Those samples were placed in RNAlater solution (ThermoFisher, USA) prior to storage at −80°C.

### 2.2 DNA and RNA extraction and sequencing

For samples collected from the native range, DNA was extracted using Qiagen DNeasy Blood and Tissue kit (Qiagen, USA) or a Serapure Bead extraction method (Rohland & Reich, 2012). For samples collected in Europe, the DNA was extracted using the NucleoSpin Tissue Kit (Macherey and Nagel, Germany). The RNA was extracted from spleen and liver samples using RNAzol RT according to the manufacturer's instructions. The quality of RNA samples was verified using Aglient Bioanalyzer (Agilent, USA), and the concentration was ascertained using fluorometer Qubit 2.0 (Thermofisher, USA). Libraries were prepared with the Illumina NEXTFlex Rapid RNA kit (Illumina, USA) and evaluated with the Aglient Bioanalyzer. The libraries were quantified using fluorometer Qubit 2.0. and then normalised to 20 nM. The Illumina NextSeq 500 v2 Mid Output Kit was used to generate clusters on the Illumina Flowcell, and the hybridised libraries were sequenced on one lane of a Flowcell on the Hiseq4000 with 2 × 150 cycles of a paired‐end sequencing module.

### 2.3 MIP design

We developed Molecular Inversion Probes (MIPs; Niedzicka et al., 2016) to perform a targeted sequencing of many genes of interest in several populations. The MIPs enable the sequencing of short ~112‐bp fragments that cover coding parts of genes. Detailed methods on the MIP design are described in the Supplementary Material. To design the MIPs, de novo transcriptome assembly was performed from four individuals from the Polish population using Trinity v2.0.4 (Grabherr et al., 2011). Nonredundant transcript open reading frames were searched for the gene orthologues in the ferret (MusPutFur1.0, Ensembl v88) and a dog (CanFam3.1, Ensembl v88) reference genomes using the reciprocal best hit, giving a total 12,477 orthologues. Reads of the four individuals were subsequently mapped onto the transcriptome to identify SNPs that could be used in MIP design to avoid excessive variation inside probe sequences. Reads were mapped with bowtie2 v2.2.4 (Langmead & Salzberg, 2012) giving an average per‐sample read depth of 350× and standard deviation of 24.9×, and SNPs were called with GATK's v3.7 *UnifiedGenotyper* (DePristo et al., 2011). The exon–intron boundaries were determined based on the ferret and dog gene models, and the corresponding exon coordinates downloaded from Ensembl v88. Transcripts were aligned to gene models of ferret/dog orthologues and exon–intron boundaries were transferred onto raccoon transcripts. The MIPs were designed using the *mipgen* program (https://github.com/shendurelab/MIPGEN, Boyle et al., 2014), such that they did not overlap with exon–intron boundaries and regions with more than one polymorphic site. We selected the MIPs targeting partial sequences of 246 immune genes, for which we could capture synonymous and nonsynonymous genetic diversity. The set included toll‐like receptor genes, MICA and MICB genes (MHC class I homologues), cytokines, genes of the complement system and genes known to be associated with helminth infection (Fumagalli et al., 2010). While referring to the specific genes, we used the names of the ferret or dog orthologue genes in cases where a ferret orthologue was unavailable.

### 2.4 MIPs resequencing

Target capture and library construction were performed using the protocol described in Hiatt et al. (2013) with modifications during the library amplification. The probes were pooled equimolarly and 5′‐phosphorylation was performed using 85 μl of the pool, 50 units of T4 Polynucleotide Kinase (NEB, USA) and 10 μl of 10 × T4 DNA ligase buffer in a total volume of 100 μl. The reaction was incubated for 45 min at 37°C, followed by the inactivation of the kinase at 80°C for 20 min. Captures were performed using 300–500 ng of genomic DNA, the phosphorylated probe pool at a 1000‐fold probe‐to‐target molar excess (adjusted for the rebalanced pool, see below), and 1 μl of 10 × Ampligase DNA ligase buffer (Epicentre, USA) in a total volume of 10 μl. The hybridisation mixture was incubated at 98 °C for 3 min, 85°C for 30 min, 60°C for 60 min and 56°C for 120 min. Gap filling and ligation reactions contained 10 μl of the hybridisation mixture, 300 pm of each dNTPs (NEB), 20 nm NAD^+^ (NEB), 7.5 μM betaine (Sigma), 1 μl of 10 × Ampligase DNA ligase buffer, five units of Ampligase DNA ligase (Epicentre) and 3.2 units of Phusion DNA polymerase (NEB) in a total volume of 20 μl and were carried out at 56°C for 60 min and 72°C for 20 min. The reactions were then cooled to 37°C and 20 units of Exonuclease I (NEB) and 100 units of Exonuclease III (NEB) were added to degrade uncircularised probes and genomic DNA. The reactions were incubated at 37°C for 45 min and at 80°C for 20 min. PCR amplification of captured targets was performed for each sample using 25 μl of Multiplex PCR Kit (Qiagen), 0.5 μM of each indexed primer, 5 μl of capture reaction and nuclease‐free water to 50 μl. The following PCR conditions were used: 95°C/15 min, 28x (94°C/30 s, 65°C/90 s, 72°C/90 s), 72°C/10 min. The PCR products from multiple samples were pooled equimolarly, run on a 1.5% agarose gel at 6.5 V/cm for 60 min and the band at ca. 270 bp was excised and purified using MinElute Gel Extraction Kit (Qiagen). The purified PCR product was quantified via Qubit (Thermofisher) and run on a Bioanalyzer (Agilent) to check the quality of the library. The library was then diluted to 12 pM and sequenced using custom primers (the primer sequences will be deposited in the Dryad repository upon publication) on the MiSeq platform, producing 2 × 150 bp paired‐end reads. We first run the experiment for four individuals applying different DNA and MIPs concentrations and adjusted the concentrations accordingly while running the whole dataset to obtain the minimal coverage of 12x per MIP.

### 2.5 Analysis of MIP targets

Details of the analysis of MIP targets are provided in the Supplementary Material. In brief, using *bwa‐mips* mapper (https://github.com/brentp/bwa‐mips) to remove MIP primer sequences (arms) of all sequenced raccoon individuals (*n* = 442) were mapped individually to the 246 transcripts used to design the MIPs. Coverage was estimated using *samtools* v1.9 *bedcov* (Li et al., 2009). Mean read depth per sample was 23.5× and standard deviation was 9.5×. Variants were initially genotyped with *GATK* v4.0.11 *HaplotypeCaller* (Poplin et al., 2018) and *samtools* v1.9/*bcftools* v1.9 with base recalibration step using *GATK BaseRecalibrator*, where SNPs discovered simultaneously by the two genotyping methods were used as reference SNPs. After recalibration, variants were genotyped jointly with *GATK GenotypeGVCFs*. Five samples with over 50% of missing data were removed. The positions with GQ <20 and DP <10 within a sample were not called. Fasta sequences were generated with *seqtk* v1.3 (https://github.com/lh3/seqtk) with *mutfa* option. A *vcf* file with biallelic SNPs was generated at the same time. The filtering was performed with *Vcftools* v0.1.16 using the following criteria: genotyping quality GQ ≥80, sequencing depth DP ≥10 and at most 15% of missing genotypes. Additional filtering was applied for *pcadapt* and *FLK* selection tests to eliminate rare variants (MAC ≥3). The gene fragments with SNPs in which observed heterozygosities reached over 0.9 in the whole dataset were removed as putative duplicates. From the final set of MIPs we filtered out the gene fragments for which the available information was insufficient to obtain legitimate estimates of Tajima's *D* and *π*, that is, less than 150 bp of a gene and containing less than three polymorphic positions. Although the designed MIPs in most cases did not cover the whole coding sequence of a gene, for simplicity we use the name ‘genes’ for the gene fragments located in specific genes throughout the text.

Most statistical analyses were performed within the R software environment (R Development Core Team, 2014). The custom scripts are available at http://github.com/konopinski/raccoon/. Due to the high level of variation and a limited number of samples from the FL population, we could not accurately reconstruct the haplotypes in this population. Therefore, our analyses were restricted only to those that did not require haplotype definitions. The data were imported to R in two different ways. For analyses on discrete SNPs (*FLK*, *pcadapt* and *DAPC*) the data were imported via *read.vcfR* function from *vcfR* package (Knaus & Grünwald, 2017) and converted to *genlight* object (Jombart, 2008) using *vcfR2genlight* function. Nonbiallelic sites were thus removed since *genlight* contains only biallelic sites. To estimate nucleotide diversity (*π*) and Tajima's *D* the data were imported via a modified *readData* function from *PopGenome* package (Pfeifer et al., 2014). The function was modified to work on the data format we used.

### 2.6 Population structure

To visualise the population structure and to check if genetic clusters detected by microsatellite and MHC loci were also mirrored in immune exonic SNPs, we used a discriminant analysis of principal components (DAPC) implemented in the R package *adegenet* (Jombart et al., 2010). Multivariate analyses are the method of choice when the assumptions of Hardy–Weinberg and linkage equilibrium within populations are violated, which might be the case for the genes under selection. First, using the function *find.clusters*, we identified the number of clusters that best reflects the genetic structuring in the data without a prior assignment of samples to given populations, using BIC scores (Bayesian Information Criterion; Jombart et al., 2010). A cross‐validation function (*Xval*.*Dapc*) was used to select the optimal number of principal components to be retained. The cross‐validation was performed with the following options: n.pca.max = 80, training.set = 0.8, result = ‘groupMean’ and n.rep = 14,000. The results of the DAPC were plotted using *scatter* function, with a population as a grouping factor.

### 2.7 Population diversity and signatures of selection

The effects of mutations (synonymous vs. nonsynonymous) were established using the *set.synnonsyn* function from the *PopGenome* (Pfeifer et al., 2014) package using standard genetic code. Using the *F.ST* function from *PopGenome* package, we calculated the nucleotide diversity (*π*) and Tajima's *D* separately for nonsynonymous and synonymous codons per each genetic cluster. The mean values of *π* and Tajima's *D* were compared between clusters using Wilcoxon matched‐pair test in *Statistica* 13.1 software (Statsoft). To search for selection footprints and/or signals of demographic events in specific genes, we used classical tests based on the frequency spectrum of mutations, as advised by Ramírez‐Soriano et al. (2008). These types of tests are more powerful when the recombination rate is unknown. Tajima's *D* test takes into account the average pairwise nucleotide diversity between sequences (*π*) and the population nucleotide diversity parameter (Watterson's *θ*_w_) that is expected under neutrality from the total number of segregating sites for a population at mutation‐drift equilibrium (Tajima, 1989), with positive values (when *π* > *θ*_w_) indicating an excess of intermediate frequency polymorphisms and negative values indicating an excess of rare polymorphisms. The values for each gene in each genetic cluster were calculated separately for nonsynonymous and synonymous substitutions using *neutrality.stats* function of the *PopGenome*. The distributions of Tajima's *D* were visualised as density plots produced using the *ggplot2* R package (Wickham, 2016).

In general, positive selection could result in a loss of genetic diversity and excess of rare variants, which can be detected by negative values of Tajima's *D*, while balancing selection resulting in an excess of intermediate frequency variants is indicated by positive Tajima's *D* values. The departure from the neutral allele frequencies can also result from population history that may alter the outcome of these statistics. In particular, positive values of the statistics are expected under a scenario of population contraction, while negative values are related to population expansion. One possibility to circumvent this problem is to acknowledge the fact that selection acts ‘locus‐wise’, while demographic processes affect all loci similarly leaving a comparable outcome across the majority of the studied genes (Stajich & Hahn, 2005). Large, multilocus dataset, consisting of immune exon‐located SNPs, and therefore under selective constraints as involved in key biological processes (Glinka et al., 2003; Wall et al., 2002) form a good dataset to be used in finding specific targets of selection in a species with little available information on its genomic architecture and selection targets. Additionally, it has been shown that the use of exonic SNPs is more powerful in resolving a population's structure than neutral markers in populations with a high level of admixture (Zhan et al., 2015). We calculated the Tajima's *D* values separately for each locus and the locus‐specific result does not depend on the remaining genes in the dataset. The deviation from neutrality is indicated by Tajima's *D* value differing from 0, but the values of *D* > 1 or *D* < ‐1 are usually regarded as indicative of selection (Ochola et al., 2010; Qiu et al., 2017). In this study, we selected genes with *D* > 1.5 on *D* < ‐1.5. Since our whole dataset consisted of exonic SNPs, we choose only genes that showed an explicitly outlier pattern. To track the patterns of gene diversity across the populations and search for genes with extreme values of the tests we calculated the 5th and 95th percentile values of synonymous and nonsynonymous Tajima's *D* values and *π* statistics for each gene in each genetic cluster. Next, we assessed whether Tajima's *D* significantly differed from values observed under neutral expectations by comparing the actual values with those resulting from 9999 coalescent simulations performed in *ms* software (Hudson, 2002) called by the *MS* wrapper function from the *PopGenome* package. For each gene in the dataset *ms* program simulates sequence polymorphisms (number, frequency and states of the polymorphic sites) assuming neutral evolution of the sequence, and the Tajima's *theta* derived from the actual polymorphism of the given gene. Tajima's *D* is then calculated for each coalescent simulation to create a null distribution of *D*. The statistical significance of the difference between the observed Tajima's *D* and zero is estimated as a proportion of Tajima's *D* estimates higher (for positive observed values) or smaller (for negative observed values) than the observed value. To identify the genes showing signs of balancing selection, we used the following criteria: at least two out of three signs of elevated Tajima's *D* value (*D* > 1.5 or *D* in the 95th percentile of highest values or a value of *D* significantly higher from neutral) in one of the three genetic clusters and at least one sign in one or both remaining clusters. Additionally, we checked if those genes have *π* values being in 95th percentile of all values. Considering the different demographic histories of the studied genetic clusters, we wanted to identify a visible trend in specific genes and therefore did not adopt stricter criteria, while identifying genes under balancing selection. To identify the genes putatively under purifying or directional selection, we employed the corresponding criteria but for negative Tajima's *D* values (Tajima's *D* values being in the 5th percentile, *D* < −1.5, Tajima's *D* value significantly lower than expected under neutrality). We used the Mann–Whitney U test in Statistica 13.1 software (Statsoft) to compare the mean level of nucleotide diversity between the genes selected using the above criteria and the remaining genes.

### 2.8 Tests for local adaptations

Two outlier detection methods were used based on different principles: *pcadapt* (Luu et al., 2017) and *FLK* (Bonhomme et al., 2010). The first method uses principal component analysis (PCA) to determine the pairwise relations between individual genotypes and searches for outlier loci that do not fit the general pattern resulting from the PCA. This method is particularly effective in populations that undergo range expansion (Luu et al., 2017). The software transforms PCA results to a set of Mahalanobis distances for each SNP site and uses the distance matrix to detect outliers. Finally, the false discovery rate (FDR) filters out sites that only differ from the bulk pattern by chance. The analysis was performed using version 4.3.3 of the R package *pcadapt* (Privé et al., 2020). We assessed the optimal principal component used in the test (K), within a range from 1 to 20 groups, by visually examining a scree plot of eigenvalues. The FDR was assessed with the *p.adjust* function with α = 0.05 (i.e. 5% of the false positives).

The second method, *FLK*, uses a phylogenetic tree that reconstructs the historical relations between populations and finds outlier sites putatively under selection pressures. The *FLK* test identifies SNPs under selection by comparing actual frequency distribution between populations and the theoretical distribution concordant with the population structure established using Reynolds' distance (Reynolds et al., 1983). We adopted this approach because of the high differentiation previously detected between the native population from FL and the European population and between the CZ population and other European populations (from Germany and Poland, Biedrzycka et al., 2014). The *FLK* approach also yielded the lowest rate of false positives among all other methods used for outlier detection (Lotterhos & Whitlock, 2014). To create a coancestry matrix, we used a set of nine putatively neutral microsatellite loci that had previously been genotyped in the studied populations (Biedrzycka et al., 2019). Although the number of loci was relatively low, the number of individual genotypes used to calculate the distance was large, while the microsatellite loci used comprised three to 20 alleles each contributing to the distance estimates, thus reducing the possible variance of the estimate, and, making it a legitimate proxy of a neutral distance between the clusters. We first computed Reynolds' genetic distance from microsatellite data using Arleqiun 3.5.2.2 (Excoffier & Lischer, 2010) and built a rooted neighbour‐joining tree using one of the populations as an outgroup. Then the FLK test statistic was computed. To detect outlier loci, we constructed a null distribution of FLK statistics under the hypothesis of neutral evolution in the *FLKnull* program (Bonhomme et al., 2010). The continuous 99% confidence intervals were estimated from 1,000,000 replicates by interpolating the results with *splinefun* function from *stats* R package. FLK test statistics above 0.995 quantiles were considered significant. Because the distances among the three clusters were roughly equal and due to lack of an outgroup, the tree could not be rooted; we therefore ran the *FLK* three times, using one cluster as an outgroup and detecting outlier loci between the two remaining clusters. First, we searched for outliers between FL and CE using CZ as an outgroup; then between CZ and FL using CE as an outgroup, and between CZ and CE using FL as an outgroup.

## 3 RESULTS

### 3.1 MiP resequencing and overall genetic diversity

A total of 1975 MIPs (~112 bp fragments) were sequenced covering the coding regions of 246 genes with variants segregating across four transcriptomes from the Polish population. Following the filtering, the dataset for the estimation of Tajima's *D* and *π* consisted of 110 genes which totalled 83,391 bp with 613 SNPs across 87 individuals: 27 from FL, 30 from CZ and 30 from CE. Filtration of SNPs in the *vcf* file for the analysis of local adaptations with MAC = 3 resulted in a dataset consisting of 428 SNP loci in 158 genes in the same 87 individuals. The number of SNPs in the analysed fragments ranged from 3 to 23 with a median of 4, while the length of the analysed genes ranged from 167 to 2053 bp with a median of 660 bp. According to the results of the DAPC, the individuals were clustered into three different groups: the native cluster from Florida (FL), the cluster from the Czech Republic (CZ) and the cluster from Germany and Poland (CE, Figure 1). The proportion of polymorphic genes in the FL population was 98.2%. In contrast, in invasive populations from Europe, the value ranged from 90% in the CE to 76.4% in the CZ populations. Similarly, the percentage of SNPs located within those genes was 76.1% in FL, but was only 44.5 and 30% in CE and CZ populations respectively (Table 1).

**TABLE 1:** Population diversity parameters for the native and invasive populations of raccoon.

	*N*	Polymorphic SNPs	Polymorphic genes	Tajima's *D* syn	Tajima's *D* NS	*π*Syn	*π*NS
FL	27	76.1%	98.1%	−0.4044 (0.82668)	−0.4568 (0.93030)	0.00065 (0.000577)	0.00053 (0.000817)
CE	30	44.5%	90.0%	−0.0026 (0.83217)	0.2390 (1.01521)	0.00050 (0.000591)	0.00049 (0.000678)
CZ	30	30.0%	76.4%	0.6147 (0.97675)	0.3981 (1.15200)	0.00043 (0.000664)	0.00045 (0.000818)

*Note*: *N* – number of individuals, Polymorphic SNPs and Polymorphic genes – within the population percentage of polymorphic SNP loci and genes respectively, Tajima's *D* Syn and NS – average Tajima's *D* within populations estimated for synonymous and nonsynonymous sites, *π*Syn and *π*NS – synonymous and nonsynonymous nucleotide diversities respectively. Standard deviations are given in the parenthesis.

The average synonymous nucleotide diversity (*π*) was the highest in the FL, moderate in the CE and the lowest for the CZ cluster (Table 1). The synonymous nucleotide diversity in the FL population was significantly higher than in both CE (Wilcoxon matched‐pairs test, *p* = 0.00023) and CZ (*p* = 0.00002). There was no difference between the CZ and CE clusters in the synonymous *π* (*p* = 0.149). Nonsynonymous nucleotide diversity was again the highest in FL, intermediate in CE and the lowest in CZ. Only the difference between FL and CZ clusters was statistically significant (Wilcoxon matched‐pairs test, *p* = 0.0021), while the differences between the CE and CZ and between the FL and CE clusters were not significant (*p* = 0.197 and *p* = 0.273 respectively).

### 3.2 Signatures of selection in site frequency spectra

**FIGURE 2:**
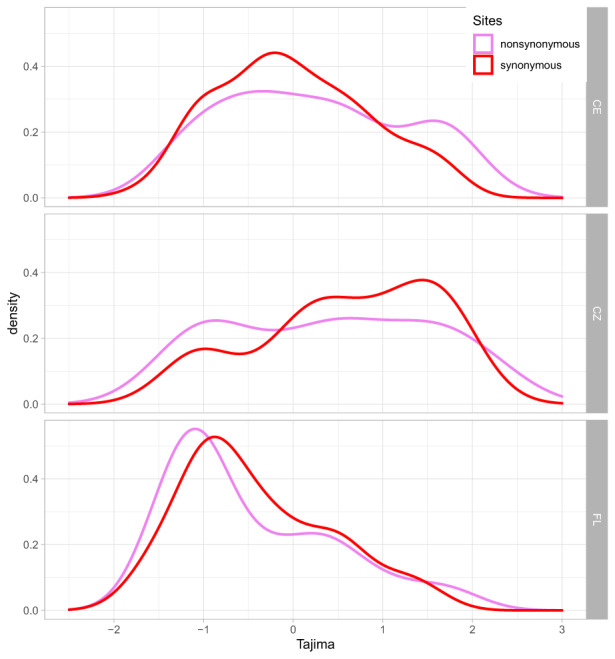
The density plots of synonymous (red) and nonsynonymous (pink) Tajima's *D* values in three genetic clusters: (a) German/Polish – CE, (b) Czech – CZ and (c) Florida – FL clusters.

The mean values of Tajima's *D* were close to neutrality for synonymous and nonsynonymous values in all the three genetic clusters (Table 1). The values were negative for the native cluster and positive in both invasive clusters (Figure 2). The difference in synonymous Tajima's *D* values was statistically significant for all pairwise comparisons, that is, Wilcoxon matched pair test *p*‐values were CZ versus FL: *p* = 0.000002, CE versus CZ: *p* = 0.00029 and CE versus FL: *p* = 0.012. Nonsynonymous Tajima's *D* values were also significantly higher in CZ than in FL (*p* = 0.00016) and in CE than in FL (*p* = 0.00020). At the same time, the difference between the CE and CZ clusters was not statistically significant for nonsynonymous *D* values (*p* = 0.413).

**FIGURE 3:**
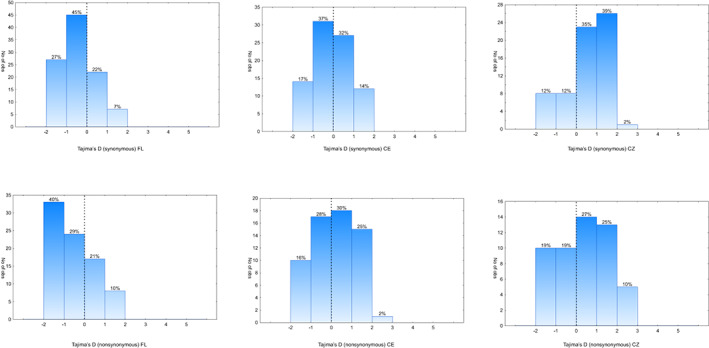
The distribution of synonymous and nonsynonymous Tajima's *D* values in three genetic clusters: German/Polish – CE, Czech – CZ and Florida – FL.

The distribution of the Tajima's *D* values in the FL cluster showed that the majority of genes (62% for synonymous and 50% for nonsynonymous substitutions) had values between −1 and 1, showing no deviation from neutrality. The percentage of genes with *D* < −1 (27% for synonymous and 40% for nonsynonymous changes) was much higher than the percentage of genes with *D* > 1 (7% for synonymous and 10% for nonsynonymous substitutions). In the CE cluster, Tajima's *D* values showed no deviation from neutrality (−1 < *D* < 1) for 69% of the genes with synonymous and 58% with nonsynonymous substitutions. In contrast to the native FL population, there were more Tajima's *D >* 1 than *D* < ‐1 (27% vs. 16%) for nonsynonymous substitutions. For synonymous substitutions, the fraction of genes with *D* > 1 and *D* < −1 was similar, as 14% and 17% respectively. In the CZ cluster, a high percentage of genes with *D* > 1 was even more pronounced and visible for both types of substitutions (41% vs. 12% for synonymous and 35% vs. 19% for nonsynonymous substitutions; Figure 3).

### 3.3 Gene‐specific patterns

**FIGURE 4:**
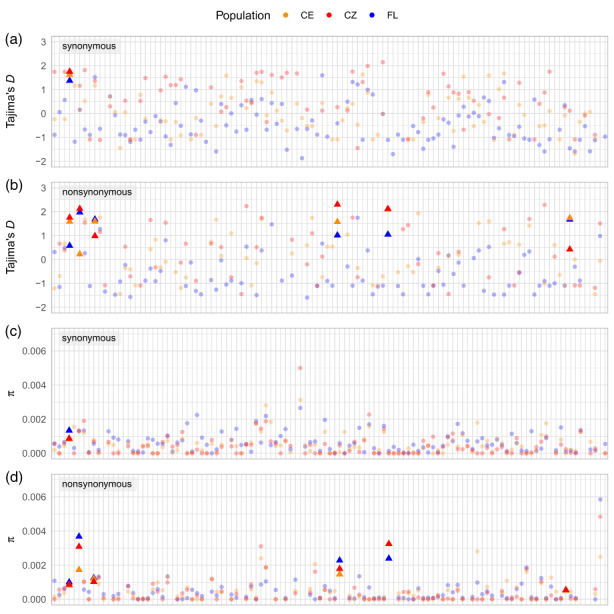
Tajima's *D* (a,b) and nucleotide diversity (c,d) values in 110 analysed genes in nonsynonymous (a,c) and synonymous (b,d) sites in the three genetic clusters: CE (orange), CZ (red) and FL (blue). The genes showing a diversity pattern resembling balancing selection in at least two genetic clusters are shown with triangles; the remaining genes are shown with circles. The list of genes putatively under balancing selection along with Tajima's *D* and *π* values are presented in Table S1.

**FIGURE 5:**
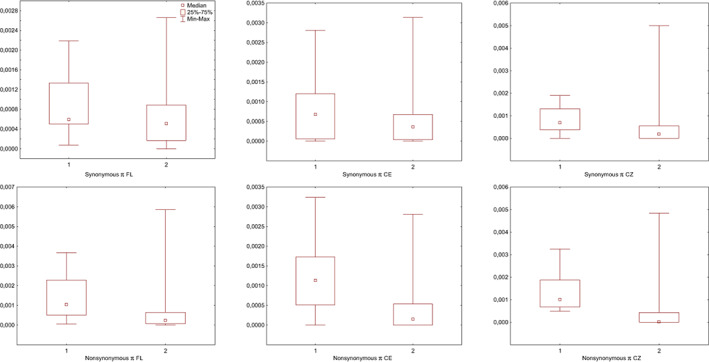
The comparison of the mean level of synonymous and nonsynonymous nucleotide diversity in three genetic clusters: German/Polish – CE, Czech – CZ and Florida – FL, between genes showing the diversity pattern resembling balancing selection (1) in at least two genetic clusters and the remaining genes in the dataset (2).

We identified the genes that exhibited outstandingly high values of nonsynonymous or synonymous Tajima's *D* values as potential targets of balancing selection. Six genes TLR8, C5AR2, TLR10, TLR1, SEMA5B and SIGLEC1 fulfilled our criteria (Table 2, Figure 4a–d). In five of the six genes the pattern was found in nonsynonymous diversity, while in the remaining one (SEMA5B), it was observed in both types of substitutions. Those genes were characterised by high *π* values, being in 95th percentile of all values for three genes and close to this boundary at either nonsynonymous or synonymous changes for the rest (Table 2, Figure 4a–d). They also showed significantly higher values of nonsynonymous nucleotide diversity relative to the rest of the dataset (Mann–Whitney *U* test *p* < 0.0046 for FL, *p* < 0.0015 for CE and *p* < 0.00001 for CZ, Figure 5) in agreement with the typical diversity patterns of balancing selection. The same comparison made for synonymous *π* values showed a significant difference only within the CZ population (*p* < 0.0104, Figure 5).

**TABLE 2:** Genes showing diversity pattern indicative of selection.

	Synonymous Tajima's *D*			Nonsynonymous Tajima's *D*			Synonymous π			Nonsynonymous π		
	FL	CE	CZ	FL	CE	CZ	FL	CE	CZ	FL	CE	CZ
TLR1	−1456^c^		0.123	1674^a^ ^,^ ^b^	1738^a^ ^,^ ^b^	0.426	0.000	0.000	0.000	0.001	0.001	0.001
SIGLEC1	−1103^c^			1049	2098^a^ ^,^ ^b^ ^,^ ^c^	2123^a^ ^,^ ^c^	0.000	0.000	0.000	0.002	*0.003*	*0.003*
TLR10	−0.407	−1084	0.269	1015	1577	2303^a^ ^,^ ^b^ ^,^ ^c^	0.001	0.000	0.001	0.002	0.002	0.002
C5AR2	0.157	0,164	1166	1979^a^ ^,^ ^b^ ^,^ ^c^	0.227	2126^a^ ^,^ ^c^	0.001	*0.001*	*0.001*	*0.004*	0.0017	0.003
SEMA5B	1378	1.627^a^	1.756^a^	0.577	1.594^a^	1756^a^ ^,^ ^b^	*0.001*	0.001	0.001	0.00010	0.001	0.001
TLR8	1526^a^ ^,^ ^b^	1176	1370	1681^a^ ^,^ ^b^	1.601^a^	0.985	0.001	0.001	0.001	0.001	0.001	0.001
ACKR1	−1587^a^ ^,^ ^c^	−1683^a^ ^,^ ^c^	−1084^b^	−0.301	0.002	−1084	0.000	0.000	0.000	0.000	0.001	0.000
CYSLTR2	−1114^c^	−1082	−0,891		−1448^b^ ^,^ ^c^	−1185^b^ ^,^ ^c^	0.000	0.000	0.000	0.000	0.000	0.001

^a^ *D* > 1.5 or *D* < −1.5.

^b^ *D* in the 95th or in the 5th percentile of all values.

^c^ Statistically different from neutrality, italicised *π* values – in the 95th percentile of all values.

Applying the established criteria for negative Tajima's *D* values, we could identify only two genes (ACKR1 and CYSLTR2) that exhibited a pattern suggestive of directional selection (Table S1). In ACKR1, the pattern was detected in synonymous diversity, while in CYSLTR2, negative Tajima's *D* values were detected both for synonymous and nonsynonymous diversity (Table 2).

Considering that directional selection may work differently depending on environmental conditions, we also searched for genes that showed lowered values of Tajima's *D* in only one of the clusters. According to this criterion, we found additional genes with nonsynonymous Tajma's *D* values located in the 5th percentile: XCR1, TLR2, TAL1, IL9R in the FL cluster; CD101 in the CE cluster; PDGFRB and NLRP1 in the CZ cluster. For synonymous Tajima's *D* values, this criterion was fulfilled for: C1QC, RPS6KA2, IL4R, ACKR2, MLPH and POSTN in the FL cluster; ITGA8, SEMA3F and TLR2 in the CE cluster; and LEPR, IL1R2 and IL17RA in the CZ cluster.

We detected a high number of genes with Tajima's *D* < −1 in the FL cluster (33 for nonsynonymous and 27 for synonymous sites). In most of the cases, the value was significantly lower than expected under neutrality. Interestingly, many of those genes (27 for nonsynonymous and 14 for synonymous substitutions) were monomorphic in one or both European clusters.

### 3.4 Local adaptation tests

**FIGURE 6:**
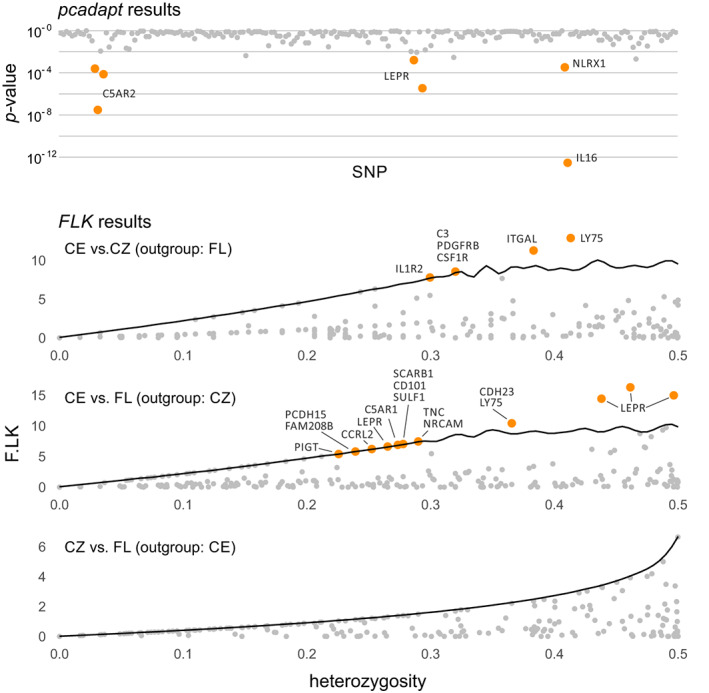
The results of local adaptation tests. (a) *p*‐values from *pcadapt*, (b–d) F.LK statistic values plotted against total heterozygosity (*H*_*T*_), solid line – 0.995 quantile of theoretical distribution of F.LK. Orange – significant values that passed the false discovery rate (FDR) correction (*pcadapt*) or are in the upper 0.995 range of theoretical distribution (*FLK*), grey – not significant.

The *pcadapt* software identified seven SNPs in four genes as being under selection (Figure 6a), that is, C5AR2 (3 SNPs), LEPR (2 SNPs), NLRX1 (1 SNP) and IL16 (1 SNP). The optimal number of principal components used in the analyses was K = 3. Only one gene possessed SNPs recognised as being under selection by both methods, i.e., LEPR, for which two outlier SNPs were detected by *pcadapt* and four by FLK. Nevertheless, only one of those SNPs was labelled by both methods. The gene C5AR2 was also selected as being under balancing selection according to the Tajma's *D* and *π* criteria.

Using FLK software, we detected 19 genes which were putatively subjected to local adaptations (Table S1, Figure 6b–d). Those genes had between one and four outlier SNPs. Most outlier SNPs (23) were found between the CE and FL clusters, with the CZ cluster used as an outgroup. Six of those SNPs (detected in genes IL1R2, C3, CSF1R, PDGFRB and ITGAL) were outliers also between the CE and CZ clusters, with the FL cluster used as an outgroup. No outlier SNPs were found between the FL and CZ populations. The gene ITGAL was also considered as being under balancing selection according to Tajma's *D* and *π* criteria.

## 4 DISCUSSION

Our results show that the invasive populations of raccoons have reduced genetic diversity compared to the native population. In the invasive populations, we observed a lower percentage of polymorphic SNPs, lower nucleotide diversity values, as well as lower gene diversity. This result is consistent with the prediction that invasive populations suffer a reduction in genetic diversity in functional, immune‐related genes due to the demographic bottleneck. The decline of genetic diversity was visible for both the synonymous and nonsynonymous diversity of the studied immune genes.

The population structure revealed by the immune genes was broadly consistent with the structure that had previously been reported on by other genetic markers (Biedrzycka et al., 2019; Fischer et al., 2017). The mixed origin of raccoon populations in Central Europe was previously confirmed through analyses of neutral microsatellite markers, mitochondrial DNA and MHC‐DRB locus. In this study, we detected a clear division between the American native FL cluster, the CZ cluster from the Czech Republic and populations from Germany and Poland (CE cluster). An admixture detected within the German and Polish raccoon populations may have been a leading cause of the relatively high levels of genetic diversity within the CE cluster, which could have facilitated the recent geographical expansion of the species.

Despite the lack of a selection signal in most studied genes, we observed clear differences in synonymous and nonsynonymous nucleotide diversities between the studied clusters. We found the highest diversity in the native FL cluster for both types of substitutions. This genetic cluster was characterised by a high effective population size and a high level of connectivity within the studied area (Troyer et al., 2014), enabling substantial gene exchange. Therefore, we treated this cluster as a proxy of a stable and panmictic population. Our result shows that in the case of raccoons, their invasive population has suffered a reduction in functional genetic diversity. Nevertheless, nucleotide diversity in the CE cluster was significantly lower than that in the FL cluster only for synonymous changes. This finding suggests that nonsynonymous substitutions, which are a primary target of selection in an invasive population that retained relatively high level of the original variation, could be maintained at a level similar to this in the native range. The lowest nucleotide diversity, which was significantly lower than in the native cluster for both synonymous and nonsynonymous diversity, was detected in the CZ cluster. This population was established from a small number of individuals that escaped from captivity and have since remained isolated from the rest of the invasive raccoon range (Biedrzycka et al., 2014). The CZ population is also around 40 years younger than the core raccoon populations from Germany and shows no signs of rapid expansion. In the case of the CZ cluster, the bottleneck may have played a primary role in shaping not only a neutral but also a functional immune genetic diversity. The CZ population was established from single source individuals (originating from captivity) and the propagule pressure (i.e. the number of introduced individuals) was low.

The effect of the demographic process was also visible in the frequency distributions of nucleotide substitutions that were different in each cluster. There were clear differences in the percentage of genes with an excess of rare and intermediate frequency variants between the clusters. In the native cluster, we observed four times more genes (7% of genes with Tajima's *D* > 1 vs. 27% with *D* < ‐1 for synonymous variants and 10 vs. 40% for nonsynonymous variants) that exhibited an excess of rare variants, a pattern suggestive of the population growth or directional selection. In the FL raccoon population, low *D* values cannot be explained by expansion since the population remains at a stable size (Troyer et al., 2014). However, most of the studied genes showed no deviation from neutrality, some genes show a pattern consistent with a balancing selection (positive values of Tajima's *D*). Therefore, strong negative values of Tajima's *D* could support the action of directional or purifying selection in a subset of genes in a native cluster. According to several genome‐wide analyses performed in humans, more than 300 genes related to immunity present signatures of positive directional selection (Barreiro & Quintana‐Murci, 2010). Comparative analyses of birds and mammal species has also shown that positive selection is the primary driver of immune genes diversity (Shultz & Sackton, 2019). Considering that immune genes are evolutionarily ancient and the pressure from parasites is constant, purifying selection is the most parsimonious way of evolution of the immune genes, especially in stable populations (Campbell et al., 2019; Mukherjee et al., 2009). The signal of purifying selection is widely visible in the innate immunity genes (Deschamps et al., 2016) that form most of our dataset.

Out of 33 genes with nonsynonymous Tajima's *D < ‐1* in the native cluster 25 were monomorphic in at least one invasive cluster (13 were monomorphic in both). In each of such cases, the SNP variant present in the invasive cluster was the most frequent one in the native population. This observation suggests that variants under directional selection in the native cluster (therefore occurring in high frequencies), were more likely to persist in the invasive populations despite the bottleneck, and/or variants kept in the native population at low frequencies (putatively due to purifying selection) were more likely to become lost during the establishment of a population outside the natural range. Although we do not have sufficient evidence, it is tempting to hypothesise that the genetic purging of less optimal variants, which in the native population were kept at low frequencies by the action of natural selection, might increase the fitness of the novel population. This explanation would only be legitimate if the positive effect of those variants in the invasive range could be determined, which we were incapable of establishing in this study.

In the CE cluster, the percentage of genes with Tajima's *D* > 1 was higher than that of genes with *D <* ‐1 for nonsynonymous changes (27 vs. 16% respectively) and comparable (14 vs. 17% respectively) for synonymous changes, resulting in a significantly higher mean population Tajima's *D* relative to the native cluster for both types of substitutions. This result, to some extent, could be an effect of mixing individuals from different introduction events (Biedrzycka et al., 2014; Fischer et al., 2017). It could also have arisen through losing rare variants as a result of a bottleneck. On the other hand, for nonsynonymous changes, the proportion of genes with an excess of balanced polymorphism (*D* > 1) was twice as high as the proportion of genes with an excess of rare variants (*D* < −1), which may be a sign of balancing selection. Nonsynonymous nucleotide diversity in the CE cluster was comparable to that of the native cluster. Despite an apparent reduction of overall genetic diversity, mixing new functional variants, possibly separated in the native range could have created a new source of variation. Such a newly created variation may have either activate the host defence against diverse pathogens and therefore been maintained in invasive populations or serve as a source of diversity on which directional selection may have acted.

The percentage of genes in the CZ cluster with Tajima's *D* > 1 was even higher than in the CE cluster (41% and 35% for synonymous and nonsynonymous substitutions respectively). This result, coupled with synonymous and nonsynonymous nucleotide diversity which were significantly lower in CZ cluster than in the native one, was most likely caused by small number of founding individuals. The CZ population is geographically separated from the main invasive range of raccoons; thus, it seems that the effect of a strong initial bottleneck in this population is still visible. This result is consistent with the previous study that used microsatellite and MHC‐DRB locus (Biedrzycka et al., 2019). Here, we also detected fewer outlier SNPs in this invasive population, as indicated by the FLK test. The effect of a strong bottleneck coupled with isolation may be the reason for a weaker response to selection in this invasive population.

### 4.1 Gene‐specific patterns

The analysis of the gene‐specific values of *π* and Tajima's *D*, suggest that most of the analysed genes followed a neutral evolution pattern. The decreased genetic diversity of the invasive clusters suggests the effect of the stochastic process. On the other hand, our results also suggest that the selection regime and the outcome of the adaptive process might have differed depending on a newly established population's demographic history and its genetic background. The action of balancing selection is supported by the presence of genes that, characterised by outstandingly high *π* and Tajima's *D* and low *F*_ *ST*_ values, present distinct patterns of genetic diversity. We selected genes exhibiting site frequency spectrum characteristic for balancing selection in at least two of the three analysed clusters. It is possible that different selective pressures may have helped to maintain several variants in average frequencies in one cluster while selecting for one specific variant in the other, or selective pressure may have been absent. Such differential selection could be possible in the invasive raccoon clusters since we detected significant differences in their parasite assemblages (Biedrzycka et al., 2020). Therefore, we applied relatively relaxed criteria in selecting genes under balancing selection, which, on the other hand, could have contributed to excluding some genes showing weaker selection patterns. In this way, we detected six genes that exhibited elevated values of Tajima's *D* and high values of nucleotide diversity.

Most genes showing the diversity pattern characteristic of balancing selection belong to toll‐like receptors (TLRs), cytokines and cytokine‐related genes involved in the inflammatory response. Three of these gene fragments are located in TLR1, TLR8 and TLR10. Toll‐like receptors are a large gene family that code for antigen‐presenting components of the innate immune response. Indeed, TLRs, acting as pattern‐recognition proteins, are expected to evolve under parasite‐driven selection promoting high variability. This group of genes has been shown to be under balancing selection in humans (Ferrer‐Admetlla et al., 2008). Balancing selection acting on TLRs, including TLR1, has previously been detected in free‐living bank vole populations. Importantly, an association between specific TLR1 alleles and infection with the blood pathogen *Bartonella* has also been identified (Kloch et al., 2018).

The signal of balancing selection detected in complement component gene C5AR2 contradicts the results of previous findings that report positive selection shaping the diversity of this highly variable innate immunity gene family (Kosiol et al., 2008; Webb et al., 2015). The complement complex is not only part of the innate immune system, but also a part of the major histocompatibility complex (Kaufman, 2022). Therefore, it may also undergo balancing selection typical for the crucial genes in this region (Hedrick, 1998). The C5AR2 gene, has previously been shown to interact with TLR4 in mice (Zhang et al., 2007) and balancing selection has also been detected in other TLR genes (Dannemann et al., 2016; Kloch et al., 2018). It is, therefore, reasonable to speculate that, from an evolutionary perspective, maintaining multiple genetic variants in average frequencies in complement genes may also be advantageous for the individuals, especially in natural habitats with high pathogen pressure.

Balancing selection pattern was also detected in semaphorin SEMA5B and endoplasmatic reticulum amino peptidase ERAP1. Semaphorin genes upregulate cytokine expression and decrease TLR expression (Iragavarapu‐Charyulu et al., 2020). Moreover, SNPs located in SEMA5A gene have been found to be associated with helminth infection in humans (Fumagalli et al., 2010). In humans, the ERAP1 gene plays a major role in regulating the repertoire of peptides presented on HLA class I alleles at the cell surface (Reeves & James, 2018), and it is thus possible, that balancing selection in raccoons is shaping the diversity of those two immune genes both in the native and invasive ranges.

Our results are consistent with the expectation that a population may benefit from standing genetic variation to cope with environmental changes quickly, that is, variants with high initial frequencies that are available immediately in the new environment (Jump et al., 2009; Lee & Gelembiuk, 2008). The selection of variants that have been present in a population creates a pattern resulting from ‘soft’ selective sweep. The ‘soft’ sweeps are believed to be the main force creating adaptive variation (Hermisson & Pennings, 2017; Przeworski et al., 2005), but detecting their footprint is problematic in terms of statistical analyses. The selective sweeps are indistinguishable from population forces such as drift using traditional methods involving linkage‐disequilibrium analysis (Jensen, 2014). Nevertheless, our results appear to agree with the prediction that a high level of genetic diversity generated by mixing phylogenetically divergent individuals in a new species range or transferred from a native range within the genes being under balancing selection (Lee & Gelembiuk, 2008) is an important factor facilitating adaptation to a novel environment.

Only two genes with a pattern that may resemble directional selection were detected in our study in two of the three clusters. The ACKR1 gene exhibited significantly negative synonymous Tajima's *D* values in the FL and CE clusters, while the CYSLTR2 gene presented a directional selection pattern of nonsynonymous diversity in the CE and CZ clusters. Considering that directional selection may favour different genetic variants in different genetic clusters, we pointed out several other genes with Tajima's *D* values being in the 5th percentile of all per‐cluster values (Table S1). Most of these genes were chemokine‐related genes, and five had previously been detected as being associated with helminth infection in human populations (Fumagalli et al., 2010). This result could confirm that the action of directional selection may overcome the effect of population expansion, which is currently ongoing in the CE cluster. Nevertheless, we are aware that very complex demographic history, including bottleneck, mixing of individuals of different phylogeographic origins and population expansion dynamics in the CE cluster may blur the footprint of possible ongoing selection.

The highest number of genes with highly negative Tajima's *D* values was found in the FL cluster both in the nonsynonymous and synonymous diversities. Because the population demography should have exerted a relatively small effect on the genetic diversity in this cluster, we treated this result with a higher level of certainty. In fact, most of these genes are involved in an ancient innate immunity response. Within the native range, one may expect that the selection has had sufficient time to favour the most efficient alleles, and the chance that newly formed variants show selective advantage should be relatively low. Therefore, purifying selection should be the main evolutionary driver of innate immune genes (Mukherjee et al., 2009).

Both *FLK* and *pcadapt* detected several SNPs putatively under selection. The SNPs detected by the two methods were mostly different which is not surprising as our dataset comprised relatively low number of SNP loci. Nevertheless, the results of both test may vary as considering that *pcadapt* detects global outliers, while *FLK* searches for outliers among defined populations. A possibility that some outliers were detected due to the random effect of genetic drift cannot be ruled out. According to the *FLK* analysis, outlier SNPs were detected in 19 gene fragments. Twelve genes were identified as possessing SNPs strongly correlated with diversity and the prevalence of common helminth infections in the analysis of HGDP‐CEPH (Human Genome Diversity Cell Line Panel) conducted among 52 worldwide human populations (Fumagalli et al., 2010). Helminth infections are one of the main selective forces in wild carnivores (Morgan et al., 2004), and raccoons suffer from frequent gastrointestinal infections (Popiołek et al., 2011). Our previous study detected associations between nematode infection and MHC variants in invasive raccoon populations (Biedrzycka et al., 2020). Finding the footprints of selection in genes potentially involved in pathogen resistance is in line with the prediction that selective processes may occur at the short timescale in a quickly expanding species when subjected to the pressure of parasites (Vatsiou et al., 2016).

Our results show the clear footprint of the demographic process visible in both invasive clusters reflecting their different demographic histories. At the same time, we could point out the genes showing distinctive diversity patterns, putatively resulting from the action of selection. Those signals were more pronounced in the invasive cluster, where relatively high levels of diversity were created due to prior population mixing. Our results show that the different demographic histories of invasive populations (genetically diverse German/Polish cluster vs. isolated and much less diverse Czech cluster) play an important role in shaping their functional genetic diversity, which may have direct effect on the success of these populations within the invasive range. This shows the importance of screening functional genetic diversity for increased accuracy in predicting invasion success.

## CONFLICT OF INTEREST

The authors declare no conflict of interest.

## Supporting information


Table S1.

Appendix S1.

## Data Availability

The raw sequence data in fastq format and vcf file used for the analysis are deposited in the Dryad Digital Repository under the following DOI: https://doi.org/10.5061/dryad.44j0zpcj1. The associated scripts have been deposited on GitHub (https://github.com/konopinski/raccoon).
