# Erratum

**DOI:** 10.1111/eva.13534

**Published:** 2023-03-22

In *Evolutionary Applications* 16:1, for the article by Konopiński et al. (2023) entitled ‘Complex patterns shape immune genes diversity during invasion of common raccoon in Europe—Selection in action despite genetic drift’ (pages 134–151), Table 2 has been corrected as follows.

**TABLE 2:** Genes showing diversity pattern indicative of selection.

	Synonymous Tajima's *D*			Nonsynonymous Tajima's *D*			Synonymous *π*			Nonsynonymous *π*		
	FL	CE	CZ	FL	CE	CZ	FL	CE	CZ	FL	CE	CZ
TLR1	−1.456^c^		0.123	1.674^a^ ^,^ ^b^	1.738^a^ ^,^ ^b^	0.426	0.000	0.000	0.000	0.001	0.001	0.001
SIGLEC1	−1.103^c^			1.049	2.098^a^ ^,^ ^b^ ^,^ ^c^	2.123^a^ ^,^ ^c^	0.000	0.000	0.000	0.002	0.003	0.003
TLR10	−0.407	−1.084	0.269	1.015	1.577	2.303^a^ ^,^ ^b^ ^,^ ^c^	0.001	0.000	0.001	0.002	0.002	0.002
C5AR2	0.157	0.164	1.166	1.979^a^ ^,^ ^b^ ^,^ ^c^	0.227	2.126^a^ ^,^ ^c^	0.001	0.001	0.001	0.004	0.0017	0.003
SEMA5B	1.378	1.627^a^	1.756^a^	0.577	1.594^a^	1.756^a^ ^,^ ^b^	0.001	0.001	0.001	0.00010	0.001	0.001
TLR8	1.526^a^ ^,^ ^b^	1.176	1.370	1.681^a^ ^,^ ^b^	1.601^a^	0.985	0.001	0.001	0.001	0.001	0.001	0.001
ACKR1	−1.587^a^ ^,^ ^c^	−1.683^a^ ^,^ ^c^	−1.084^b^	−0.301	0.002	−1.084	0.000	0.000	0.000	0.000	0.001	0.000
CYSLTR2	−1.114^c^	−1.082	−0.891		−1.448^b^ ^,^ ^c^	−1.185^b^ ^,^ ^c^	0.000	0.000	0.000	0.000	0.000	0.001

^a^ *D* > 1.5 or *D* < −1.5.

^b^ *D* in the 95th or in the 5th percentile of all values.

^c^ Statistically different from neutrality, underlined *π* values—in the 95th percentile of all values.

We apologize for the error.
