# Supplementary material for: Complex patterns shape immune genes diversity during invasion of common raccoon in Europe – Selection in action despite genetic drift
Source: Evol Appl. 2022 Dec 11;16(1):134–51. doi: 10.1111/eva.13517 (PMC9850017; doi:10.1111/eva.13517)
Supplement: Supplementary file 2 — Appendix S1. [file EVA-16-134-s002.docx]

### Supplementary Material

**MIP design & analysis**

**Transcriptome assembly**

Read duplicates were removed with prinseq-lite v0.991 [(Schmieder and Edwards 2011)](https://paperpile.com/c/G7b7qk/ao88M), and quality trimmed using Trimmomatic v0.35 [(Bolger, Lohse, and Usadel 2014)](https://paperpile.com/c/G7b7qk/1grIh) with settings LEADING:3 TRAILING:3 SLIDINGWINDOW:4:15 MINLEN:36. Reads of all samples were merged, and 50 M of left and right reads were subsampled for the assembly using seqtk v1.2 (https://github.com/lh3/seqtk). Transcripts were assembled with Trinity v2.0.4 [(Grabherr et al. 2011)](https://paperpile.com/c/G7b7qk/TcfGA). Only transcripts with FPKM > 1 and IsoPct > 1 were retained. 446 685 transcripts were searched for open reading frames (ORFs) using the TransDecoder program in Trinity, with option -m 50 for minimum aminoacid length and no strand specificity. To remove redundancy between transcripts, similar ORFs were clustered using cd-hit [(W. Li and Godzik 2006)](https://paperpile.com/c/G7b7qk/Cem6i) with minimum identity 98% (-c 0.98) and word size 8 (-n 8). This resulted in nonredundant 172 839 transcripts.

**Identifying immune genes in raccoon transcripts**

All proteins of ferret (MusPutFur1.0, ensembl v88) and dog (CanFam3.1, ensembl v88) were downloaded from BioMart ensembl. To find orthologs of these genes in the raccoon transcriptome, reciprocal blast between proteins and raccoon transcripts was performed separately for each species. For different ORFs belonging to the same transcript with a match to the same protein, the ORF with the highest bitscore was retained. Using a custom python v2.7 script only those queries were retained, which had a single hit to a subject or only one best hit among many (average bitscore across HSP > 0.8*bitscore of the second best hits). This resulted in 12 477 transcripts with annotation to either ferret or a dog.

**Designing MIPs based on raccoon transcripts**

Molecular Inversion Probes (MIPs) enable sequencing of short ~120 bp fragments that cover coding parts of genes. MIPs were designed using program mipgen (<https://github.com/shendurelab/MIPGEN>, [(Boyle et al. 2014)](https://paperpile.com/c/G7b7qk/WTpRP)) based on transcript sequences, such that they did not overlap exon-intron boundaries and regions with more than one polymorphic site.

To obtain variant information, trimmed RNA-seq reads of four samples were mapped to the transcriptome (12 477 transcripts with annotation) using bowtie2 v2.2.4 [(Langmead and Salzberg 2012)](https://paperpile.com/c/G7b7qk/xdJAI) with option --very-sensitive-local. Duplicates were removed with picard v2.10.3 (http://broadinstitute.github.io/picard/). Reads were then sorted and realigned with samtools v1.3 [(H. Li et al. 2009)](https://paperpile.com/c/G7b7qk/IuVIa) using option calmd -AEbr. SNPs were called with GATK v3.7 [(DePristo et al. 2011)](https://paperpile.com/c/G7b7qk/XYWPO) UnifiedGenotyper with the following options: -pcr_error 5.0E-3 (PCR error rate used to calculate likelihoods), -mbq 20 (minimum base mapping quality), -stand_call_conf 20.0 (minimum phred score to make a call), -maxAlleles 6 (number of alternates considered for genotyping), -out_mode EMIT_ALL_SITES, --genotyping_mode DISCOVERY. SNPs were filtered using vcftools v0.1.12b [(Danecek et al. 2011)](https://paperpile.com/c/G7b7qk/XyKjj) to remove variants with mean DP < 8, maf < 0.15 and genotypes with GQ < 30. Vcf files generated with GATK were also converted into fasta sequences using custom python v2.7 script, where in case of heterozygotes one base was randomly assigned to one of the two haplotypes, and genotypes with GQ < 20, DP < 8 or DP decreased by 80% compared to the preceding site were encoded as missing data. Fasta sequences were then adjusted to match the length of transcripts. Mstatspop v.0.998985beta (<https://github.com/CRAGENOMICA/mstatspop>) was run to estimate the number of synonymous and nonsynonymous variants in four samples.

To obtain information about exon boundaries, exon sequences and exon coordinates of transcripts, ferret and dog genes were downloaded from BioMart ensembl, combined into gene models and blasted against transcripts with scripts from <https://github.com/molecol/targeted-resequencing-with-mips>. Because transcripts lack intron sequences which prevents mipgen from identifying MIPs at the border of exons, artificial introns of 150 bp consisting of ‘N’ bases were inserted in between the exon sequences. Files with SNPs and exon coordinates were modified accordingly to match the right positions on the transcripts.

Mipgen was run with options -common_snps off -check_copy_number off -max_mip_overlap 20 -min_capture_size 120 -max_capture_size 152 -tag_sizes 0,0 -score_methodmixed -arm_length_sums 40,41,42. MIPs with score > 1.4 were selected and highly overlapping MIPs were removed.

We prioritized selection of genes by focusing on immune genes and those which show variation based on four transcriptomes. We searched for orthologs of immune genes including Toll-like receptor genes, MICA and MICB genes, cytokines, genes of the complement system and genes known to be associated with helminth infection based on human Associated gene name in ensembl. For genes which had no orthologs in human we selected raccoon orthologs with the ferret, and if absent corresponding orthologs with the dog. This resulted in 278 nonredundant raccoon transcripts. 383 additional candidate transcripts were searched based on synonymous and nonsynonymous variation. Finally based on sequence diversity in amplified regions 1757 MIPs from 221 immune transcripts and 218 MIPs from 25 additional transcripts were selected totalling to 1975 MIPs from 246 genes.

**MIPs resequencing**

DNA was dissolved in 100 ul of TE buffer. Target capture and library construction were performed using the protocol described in (Hiatt, Pritchard, Salipante, O’Roak, & Shendure, 2013) with modifications during library amplification. Probes were pooled equimolarly and 5′-phosphorylation was performed using 85 ul of the pool, 50 units of T4 Polynucleotide Kinase (NEB) and 10 ul of 10 × T4 DNA ligase buffer in a total volume of 100 ul. The reaction was incubated for 45 min at 37 °C, followed by inactivation of the kinase at 80 °C for 20 min. Captures were performed using 300–500 ng of genomic DNA, the phosphorylated probe pool at a 1000-fold probe-to-target molar excess (adjusted for the rebalanced pool, see below), and 1 ul of 10 × Ampligase DNA ligase buffer (Epicentre) in a total volume of 10 ul. The hybridization mixture was incubated at 98 °C for 3 min, 85 °C for 30 min, 60 °C for 60 min, and 56 °C for 120 min. Gap filling and ligation reactions contained 10 ul of hybridization mixture, 300 pm of each dNTPs (NEB), 20 nm NAD^+^ (NEB), 7.5 um betaine (Sigma), 1 ul of 10 × Ampligase DNA ligase buffer, 5 units of Ampligase DNA ligase (Epicentre) and 3.2 units of Phusion DNA polymerase (NEB) in a total volume of 20 ul and were carried out at 56 °C for 60 min and 72 °C for 20 min. Reactions were then cooled to 37 °C and 20 units of Exonuclease I (NEB) and 100 units of Exonuclease III (NEB) were added to degrade not circularised probes and genomic DNA. Reactions were incubated at 37 °C for 45 min and at 80 °C for 20 min. For each sample PCR amplification of captured targets was performed using 25 ul of Multiplex PCR Kit (Qiagen), 0.5 uM of each indexed primer, 5 ul of capture reaction and nuclease-free water to 50 ul. The following PCR conditions were used: 95 °C/15 min, 28x (94 °C/30 s, 65 °C/90 s, 72 °C/90 s), 72 °C/10 min. PCR products from multiple samples were pooled equimolarly, run on a 1.5% agarose gel at 6.5 V/cm for 60 min and the band at ca. 270 bp was excised and purified using MinElute Gel Extraction Kit (Qiagen). The purified PCR product was quantified via Qubit and run on a Bioanalyzer (Agilent) to check quality of the library. The library was then diluted to 12 pM and sequenced using custom primers (primer sequences will be deposited in Dryad repository upon publication) on the MiSeq platform, producing 2 × 150 bp paired-end reads. We first run the experiment for four individuals applying different DNA and MIPs concentrations and adjusted the concentrations accordingly while running the whole dataset to obtain the minimal coverage of 12x per MIP.

**Analysis of MIP targets**

Prior to mapping, a file with coordinates of MIP primers (arms) on reference was modified to match transcript sequences without inserted artificial introns. Reads were mapped to corresponding transcripts (n=246) and arms were removed using bwa-mips mapper (<https://github.com/brentp/bwa-mips>). The bwa-mips python v2.7 script was tailored such that only one arm from the single read was removed, and the rest of the read sequence was left intact regardless of whether it was overlapping with its mate or not. Bam files were sorted and then bam files of the same samples generated in four runs were merged together using samtools v1.9 [(H. Li et al. 2009)](https://paperpile.com/c/G7b7qk/IuVIa).

To investigate coverage of reads in each position of MIP targets, we calculated depth of coverage for all target sequences using samtools with option bedcov.  All sequence positions were genotyped with GATK v4.0.11 [(Poplin et al. 2018)](https://paperpile.com/c/G7b7qk/141hm) HaplotypeCaller. Base qualities were recalibrated in four rounds until convergence. In each round a common set of SNPs was selected from SNPs called with GATK HaplotypeCaller and samtools v1.9 with bcftools v1.9 (<https://github.com/samtools>), and after that base recalibration was performed using selected SNPs with GATK BaseRecalibrator. Samtools mpileup format was generated with options samtools mpileup -C50 -d 100000 -E -q 4 -g -t DP,SP,AD,ADF,ADR,INFO/AD and final SNP calling was done with bcftools with options bcftools call -mv -f GQ. HaplotypeCaller was run with option -ERC GVCF and --disable-read-filter NotDuplicateReadFilter. After the final round of base recalibration all positions were genotyped using GATK. 5 samples with a lot of missing data were removed. A file with joined and genotyped individuals was filtered such that genotypes with GQ < 20 and DP < 10 were masked, and fasta sequences were generated using seqtk v1.3 (https://github.com/lh3/seqtk) with mutfa option. At the same time a vcf file with SNPs was generated. Variants with GQ < 20 and DP < 8 were masked, SNPs with more than 15% of missing data and multiallelic SNPs were removed. Between 20 and 30 samples were selected from each population to reduce the differences between sample sizes. The final filtering was run on resulting vcf files. Vcftools v0.1.16 was used to retain variants passing given filters: MAC > 3, GQ > 95, DP > 10. Genes in which SNPs’ observed heterozygosites were over 0.9 in the whole dataset were removed as putative duplicates.

**Geographic coordinates of samples collected from European locations**

ID long lat POP

PL050-MP 17.28644 49.42205 CZ

PL069-MP 17.28593 49.42827 CZ

PL070-MP 17.28846 49.42366 CZ

PL072-MP 17.28599 49.42223 CZ

PL074-MP 17.28816 49.42958 CZ

PL076-MP 17.28122 49.42897 CZ

PL078-MP 17.28377 49.42082 CZ

PL079-MP 17.28753 49.42233 CZ

PL172-MP 17.28528 49.42454 CZ

PL173-MP 17.28997 49.42615 CZ

PL175-MP 17.28108 49.42356 CZ

PL188-MP 17.28648 49.42426 CZ

PL191-MP 17.36647 49.35866 CZ

PL192-MP 17.36611 49.35477 CZ

PL199-MP 17.28648 49.42596 CZ

PL200-MP 17.28517 49.42043 CZ

PL273-MP 17.28869 49.42125 CZ

PL274-MP 17.28812 49.42525 CZ

PL276-MP 17.36886 49.35707 CZ

PL277-MP 17.36984 49.35746 CZ

PL199 17.29365 49.48141 CZ

PL200 17.34082 49.46263 CZ

PL202 17.28397 49.49568 CZ

PL203 17.28613 49.46477 CZ

PL204 17.30954 49.45615 CZ

PL206 17.27394 49.46203 CZ

PL207 17.33922 49.47252 CZ

PL208 17.31899 49.50223 CZ

PL209 17.36826 49.47975 CZ

PL236-MP 15.07102 51.43999 CE

PL237-MP 15.07786 51.43053 CE

PL256-MP 13.73378 51.05885 CE

PL257-MP 13.73478 51.05916 CE

PL260-MP 13.73906 51.05483 CE

PL193 13.85161 51.10216 CE

PL73 14.55272 51.23181 CE

PL75 14.33134 51.15405 CE

PL79 14.27548 51.17643 CE

PL238-MP 9.476471 51.31331 CE

PL239-MP 9.476689 51.31032 CE

PL240-MP 9.476047 51.31409 CE

PL241-MP 9.476585 51.31862 CE

PL252-MP 9.476009 51.31621 CE

PL253-MP 9.476635 51.31342 CE

PL255-MP 9.476885 51.31354 CE

PL001-MP 12.58833 53.39345 CE

PL003-MP 12.58902 53.39424 CE

PL005-MP 12.58243 53.39269 CE

PL007-MP 12.58919 53.39867 CE

PL015-MP 12.58574 53.39299 CE

PL062-MP 12.58913 53.39501 CE

PL232-MP 13.18064 53.37843 CE

PL018-MP 17.28781 49.42855 CZ

PL265-MP 14.69012 52.55925 CE

PL118-MP 14.69009 52.55011 CE

PL119-MP 14.69802 52.55586 CE

PL040-MP 14.69046 52.55703 CE

PL123-MP 14.81338 52.57194 CE

PL127-MP 14.81812 52.57246 CE

PL127 14.69533 52.55349 CE

References:

[Bolger, Anthony M., Marc Lohse, and Bjoern Usadel. 2014. “Trimmomatic: A Flexible Trimmer for Illumina Sequence Data.” *Bioinformatics*  30 (15): 2114–20.](http://paperpile.com/b/G7b7qk/1grIh)

[Boyle, Evan A., Brian J. O’Roak, Beth K. Martin, Akash Kumar, and Jay Shendure. 2014. “MIPgen: Optimized Modeling and Design of Molecular Inversion Probes for Targeted Resequencing.” *Bioinformatics*  30 (18): 2670–72.](http://paperpile.com/b/G7b7qk/WTpRP)

[Danecek, Petr, Adam Auton, Goncalo Abecasis, Cornelis A. Albers, Eric Banks, Mark A. DePristo, Robert E. Handsaker, et al. 2011. “The Variant Call Format and VCFtools.” *Bioinformatics*  27 (15): 2156–58.](http://paperpile.com/b/G7b7qk/XyKjj)

[DePristo, Mark A., Eric Banks, Ryan Poplin, Kiran V. Garimella, Jared R. Maguire, Christopher Hartl, Anthony A. Philippakis, et al. 2011. “A Framework for Variation Discovery and Genotyping Using next-Generation DNA Sequencing Data.” *Nature Genetics* 43 (5): 491–98.](http://paperpile.com/b/G7b7qk/XYWPO)

[Grabherr, Manfred G., Brian J. Haas, Moran Yassour, Joshua Z. Levin, Dawn A. Thompson, Ido Amit, Xian Adiconis, et al. 2011. “Full-Length Transcriptome Assembly from RNA-Seq Data without a Reference Genome.” *Nature Biotechnology* 29 (7): 644–52.](http://paperpile.com/b/G7b7qk/TcfGA)

[Langmead, Ben, and Steven L. Salzberg. 2012. “Fast Gapped-Read Alignment with Bowtie 2.” *Nature Methods* 9 (4): 357–59.](http://paperpile.com/b/G7b7qk/xdJAI)

[Li, Heng, Bob Handsaker, Alec Wysoker, Tim Fennell, Jue Ruan, Nils Homer, Gabor Marth, Goncalo Abecasis, Richard Durbin, and 1000 Genome Project Data Processing Subgroup. 2009. “The Sequence Alignment/Map Format and SAMtools.” *Bioinformatics*  25 (16): 2078–79.](http://paperpile.com/b/G7b7qk/IuVIa)

[Li, Weizhong, and Adam Godzik. 2006. “Cd-Hit: A Fast Program for Clustering and Comparing Large Sets of Protein or Nucleotide Sequences.” *Bioinformatics*  22 (13): 1658–59.](http://paperpile.com/b/G7b7qk/Cem6i)

[Poplin, Ryan, Valentin Ruano-Rubio, Mark A. DePristo, Tim J. Fennell, Mauricio O. Carneiro, Geraldine A. Van der Auwera, David E. Kling, et al. 2018. “Scaling Accurate Genetic Variant Discovery to Tens of Thousands of Samples.” *Cold Spring Harbor Laboratory*. https://doi.org/](http://paperpile.com/b/G7b7qk/141hm)[10.1101/201178](http://dx.doi.org/10.1101/201178)[.](http://paperpile.com/b/G7b7qk/141hm)

[Schmieder, Robert, and Robert Edwards. 2011. “Quality Control and Preprocessing of Metagenomic Datasets.” *Bioinformatics*  27 (6): 863–64.](http://paperpile.com/b/G7b7qk/ao88M)
